# *N*-Ethyl-*N*-phenyl-*N*′-tosyl­formamidine

**DOI:** 10.1107/S1600536809021953

**Published:** 2009-06-17

**Authors:** Heng-Shui Shen, Nan Liu, Zi-Cheng Li, Wen-Cai Huang

**Affiliations:** a Key Laboratory of Drug Targeting and Drug-Delivery Systems of the Ministry of Education, Department of Medicinal Chemistry, West China School of Pharmacy, Sichuan University, Chengdu 610041, People’s Republic of China; b Department of Pharmaceutical and Bioengineering, School of Chemical Engineering, Sichuan University, Chengdu 610065, People’s Republic of China

## Abstract

The title compound, C_16_H_18_N_2_O_2_S, was obtained as an unexpected product while attempting to form carbon–nitro­gen bonds by catalytic amidation. The mol­ecule displays an *E* conformation about the C=N double bond. The planes of the two aromatic rings in the mol­ecule form a dihedral angle of 47.06 (9)°.

## Related literature

For the crystal structures of related compounds, see: Cole *et al.* (2005 ▶, 2007 ▶). For the synthesis of substituted sulfanilamides by catalytic amidation, see: Liu *et al.* (2008 ▶); Xu *et al.* (2007 ▶, 2008 ▶).
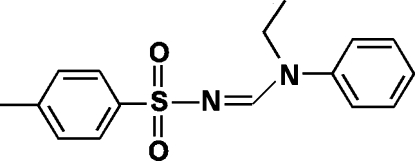


## Experimental

### 

#### Crystal data

C_16_H_18_N_2_O_2_S
*M* *_r_* = 302.38
Monoclinic, 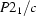

*a* = 16.306 (5) Å
*b* = 8.122 (4) Å
*c* = 12.674 (4) Å
β = 108.22 (2)°
*V* = 1594.3 (10) Å^3^
*Z* = 4
Mo *K*α radiation
μ = 0.21 mm^−1^
*T* = 291 K
0.60 × 0.46 × 0.42 mm

#### Data collection

Enraf–Nonius CAD-4 diffractometer
Absorption correction: spherical (*WinGX*; Farrugia, 1999 ▶) *T*_min_ = 0.885, *T*_max_ = 0.918
3769 measured reflections
2928 independent reflections
1958 reflections with *I* > 2σ(*I*)
*R*_int_ = 0.005
3 standard reflections every 200 reflections intensity decay: 2.7%

#### Refinement

*R*[*F* ^2^ > 2σ(*F* ^2^)] = 0.054
*wR*(*F* ^2^) = 0.154
*S* = 1.09
2928 reflections
192 parameters
H-atom parameters constrained
Δρ_max_ = 0.34 e Å^−3^
Δρ_min_ = −0.44 e Å^−3^

### 

Data collection: *DIFRAC* (Gabe & White, 1993 ▶); cell refinement: *DIFRAC*; data reduction: *NRCVAX* (Gabe *et al.*, 1989 ▶); program(s) used to solve structure: *SHELXS97* (Sheldrick, 2008 ▶); program(s) used to refine structure: *SHELXL97* (Sheldrick, 2008 ▶); molecular graphics: *ORTEP-3 for Windows* (Farrugia, 1997 ▶); software used to prepare material for publication: *SHELXL97*.

## Supplementary Material

Crystal structure: contains datablocks global, I. DOI: 10.1107/S1600536809021953/rz2331sup1.cif

Structure factors: contains datablocks I. DOI: 10.1107/S1600536809021953/rz2331Isup2.hkl

Additional supplementary materials:  crystallographic information; 3D view; checkCIF report

## Figures and Tables

**Fig. 1.**
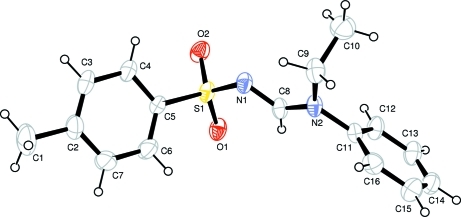
The molecular structure of the title compound, with displacement ellipsoids drawn at the 50% probability level.

**Fig. 2.**
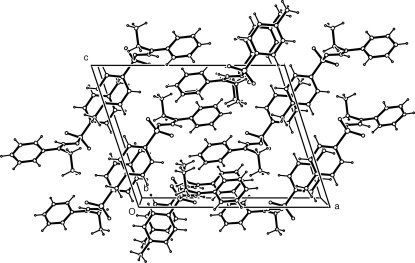
A packing diagram of the title compound approximately viewed along the b axis.

## supplementary crystallographic information

### Comment

In the course of our studies aimed to prepare a substituted sulfanilamide from the corresponding tertiary amines by catalytic amidation using a transition metal salt (Xu *et al.*, 2008; Xu *et al.*, 2007; Liu *et al.*, 2008), the title compound was unexpectedly obtained in about 54% yield.

The molecule of the title compound (Fig. 1) dispays an *E* conformation about the C8═N1 double bond. The values of the N1-C8 (1.301 (3) Å) and N2-C8 (1.326 (3) Å) bonds indicate some degree of conjugation, which was not observed in the related compounds N,N'-bis(2,6-diisopropylphenyl)-N-(4-(3',4',5'-trifluorophenoxy)butyl)formamidine (Cole *et al.*, 2007) and N-(4-(2,3,4,5-tetrafluorophenoxy)butyl)-N,N'-bis(2,6-diisopropylphenyl)formamidine (Cole *et al.*, 2005). The dihedral angle formed by the phenyl and benzene rings is 47.06 (9)°. The crystal structure (Fig. 2) is enforced only by van der Waals interactions.

### Experimental

*N*,*N*-Diethylaniline (149 mg, 1 mmol), *p*-toluenesulfonyl azide (591 mg, 3 mmol), copper(I) chloride(20 mg, 0.2 mmol), TEBA (triethylbenzylammonium chloride) (22.7 mg, 0.1 mmol) and acetonitrile (5 mL) were added into a 25 mL round-bottom flask. The resulting mixture was stirred and refluxed for 8 h, then it was evaporated to almost dryness under reduced pressure. Purification was performed by column chromatography on silica gel with petroleum ether/ethyl acetate (7:1–6:1, *v*/*v*) as eluent to give the pure product (163 mg, yield 54%). Colourless crystals suitable for X-ray analysis were obtained by slow evaporation of a cyclohexane/acetyl acetate solution (5:1 *v*/*v*) at room temperature.

### Refinement

H atoms were positioned geometrically and refined using a riding model, with C—H = 0.93–0.97 Å and with *U*_iso_(H) = 1.2*U*_eq_(C) or 1.5*U*_eq_(C) for methyl H atoms.

### Crystal data

**Table:**

C_16_H_18_N_2_O_2_S	*F*(000) = 640
*M**_r_* = 302.38	*D*_x_ = 1.260 Mg m^−^^3^
Monoclinic, *P*2_1_/*c*	Mo *K*α radiation, λ = 0.71073 Å
Hall symbol: -P 2ybc	Cell parameters from 25 reflections
*a* = 16.306 (5) Å	θ = 4.7–7.7°
*b* = 8.122 (4) Å	µ = 0.21 mm^−^^1^
*c* = 12.674 (4) Å	*T* = 291 K
β = 108.22 (2)°	Block, colourless
*V* = 1594.3 (10) Å^3^	0.60 × 0.46 × 0.42 mm
*Z* = 4	

### Data collection

**Table:**

Enraf–Nonius CAD-4 diffractometer	1958 reflections with *I* > 2σ(*I*)
Radiation source: fine-focus sealed tube	*R*_int_ = 0.005
graphite	θ_max_ = 25.5°, θ_min_ = 1.3°
ω–2θ scans	*h* = −6→19
Absorption correction: for a sphere (*WinGX*; Farrugia, 1999)	*k* = −9→0
*T*_min_ = 0.885, *T*_max_ = 0.918	*l* = −15→14
3769 measured reflections	3 standard reflections every 200 reflections
2928 independent reflections	intensity decay: 2.7%

### Refinement

**Table:**

Refinement on *F*^2^	Primary atom site location: structure-invariant direct methods
Least-squares matrix: full	Secondary atom site location: difference Fourier map
*R*[*F*^2^ > 2σ(*F*^2^)] = 0.054	Hydrogen site location: inferred from neighbouring sites
*wR*(*F*^2^) = 0.154	H-atom parameters constrained
*S* = 1.09	*w* = 1/[σ^2^(*F*_o_^2^) + (0.0973*P*)^2^] where *P* = (*F*_o_^2^ + 2*F*_c_^2^)/3
2928 reflections	(Δ/σ)_max_ < 0.001
192 parameters	Δρ_max_ = 0.34 e Å^−^^3^
0 restraints	Δρ_min_ = −0.44 e Å^−^^3^

### Special details

**Table:**

Geometry. All e.s.d.'s (except the e.s.d. in the dihedral angle between two l.s. planes) are estimated using the full covariance matrix. The cell e.s.d.'s are taken into account individually in the estimation of e.s.d.'s in distances, angles and torsion angles; correlations between e.s.d.'s in cell parameters are only used when they are defined by crystal symmetry. An approximate (isotropic) treatment of cell e.s.d.'s is used for estimating e.s.d.'s involving l.s. planes.
Refinement. Refinement of *F*^2^ against ALL reflections. The weighted *R*-factor *wR* and goodness of fit *S* are based on *F*^2^, conventional *R*-factors *R* are based on *F*, with *F* set to zero for negative *F*^2^. The threshold expression of *F*^2^ > σ(*F*^2^) is used only for calculating *R*-factors(gt) *etc*. and is not relevant to the choice of reflections for refinement. *R*-factors based on *F*^2^ are statistically about twice as large as those based on *F*, and *R*- factors based on ALL data will be even larger.

### Fractional atomic coordinates and isotropic or equivalent isotropic displacement parameters (Å^2^)

**Table:**

	*x*	*y*	*z*	*U*_iso_*/*U*_eq_	
S1	0.22480 (4)	0.35989 (7)	0.03686 (5)	0.0502 (2)	
O1	0.29488 (10)	0.4299 (2)	0.00582 (16)	0.0589 (5)	
O2	0.20033 (12)	0.4417 (2)	0.12215 (16)	0.0671 (5)	
N1	0.24137 (12)	0.1667 (2)	0.07283 (17)	0.0519 (5)	
N2	0.33809 (12)	−0.0487 (2)	0.10219 (16)	0.0499 (5)	
C1	−0.0882 (2)	0.3571 (5)	−0.3731 (3)	0.1032 (13)	
H1A	−0.1362	0.4031	−0.3547	0.155*	
H1B	−0.1020	0.2469	−0.4003	0.155*	
H1C	−0.0763	0.4232	−0.4294	0.155*	
C2	−0.00939 (19)	0.3541 (4)	−0.2704 (3)	0.0693 (8)	
C3	−0.01841 (18)	0.3645 (4)	−0.1664 (3)	0.0808 (9)	
H3	−0.0734	0.3725	−0.1595	0.097*	
C4	0.05244 (17)	0.3632 (4)	−0.0724 (3)	0.0706 (8)	
H4	0.0452	0.3694	−0.0026	0.085*	
C5	0.13384 (15)	0.3529 (3)	−0.0822 (2)	0.0488 (6)	
C6	0.14362 (18)	0.3421 (4)	−0.1855 (3)	0.0739 (8)	
H6	0.1985	0.3331	−0.1928	0.089*	
C7	0.0719 (2)	0.3447 (5)	−0.2784 (3)	0.0844 (10)	
H7	0.0791	0.3399	−0.3483	0.101*	
C8	0.31639 (14)	0.1071 (3)	0.07690 (19)	0.0465 (5)	
H8	0.3564	0.1767	0.0614	0.056*	
C9	0.27942 (17)	−0.1647 (3)	0.1310 (3)	0.0654 (8)	
H9A	0.2849	−0.2722	0.1006	0.078*	
H9B	0.2203	−0.1278	0.0979	0.078*	
C10	0.2990 (2)	−0.1785 (4)	0.2552 (3)	0.0852 (9)	
H10A	0.3586	−0.2073	0.2887	0.128*	
H10B	0.2631	−0.2620	0.2716	0.128*	
H10C	0.2877	−0.0749	0.2844	0.128*	
C11	0.42519 (15)	−0.1012 (3)	0.11568 (19)	0.0459 (6)	
C12	0.49282 (16)	−0.0216 (3)	0.1901 (2)	0.0597 (7)	
H12	0.4828	0.0657	0.2320	0.072*	
C13	0.57586 (17)	−0.0723 (4)	0.2023 (3)	0.0689 (8)	
H13	0.6220	−0.0188	0.2529	0.083*	
C14	0.59129 (19)	−0.1996 (4)	0.1413 (3)	0.0700 (8)	
H14	0.6477	−0.2328	0.1505	0.084*	
C15	0.5245 (2)	−0.2778 (4)	0.0673 (3)	0.0753 (9)	
H15	0.5352	−0.3637	0.0249	0.090*	
C16	0.44046 (19)	−0.2311 (3)	0.0543 (2)	0.0611 (7)	
H16	0.3946	−0.2867	0.0047	0.073*	

### Atomic displacement parameters (Å^2^)

**Table:**

	*U*^11^	*U*^22^	*U*^33^	*U*^12^	*U*^13^	*U*^23^
S1	0.0381 (3)	0.0471 (4)	0.0608 (4)	0.0024 (2)	0.0089 (3)	−0.0001 (3)
O1	0.0414 (9)	0.0505 (9)	0.0797 (12)	−0.0026 (7)	0.0118 (9)	0.0065 (9)
O2	0.0571 (11)	0.0710 (12)	0.0682 (12)	0.0060 (9)	0.0124 (9)	−0.0151 (10)
N1	0.0370 (10)	0.0499 (11)	0.0639 (13)	0.0017 (8)	0.0084 (9)	0.0074 (9)
N2	0.0409 (11)	0.0454 (11)	0.0538 (12)	0.0006 (8)	0.0010 (9)	0.0040 (9)
C1	0.067 (2)	0.126 (3)	0.089 (2)	0.013 (2)	−0.0152 (19)	−0.004 (2)
C2	0.0511 (15)	0.0738 (18)	0.0694 (19)	0.0089 (13)	−0.0011 (14)	−0.0040 (15)
C3	0.0391 (14)	0.114 (3)	0.084 (2)	0.0170 (16)	0.0112 (15)	0.0069 (18)
C4	0.0431 (14)	0.099 (2)	0.0668 (17)	0.0185 (14)	0.0139 (13)	0.0050 (16)
C5	0.0368 (12)	0.0470 (12)	0.0596 (15)	0.0059 (10)	0.0108 (11)	0.0012 (11)
C6	0.0446 (15)	0.107 (2)	0.0693 (19)	0.0033 (15)	0.0162 (14)	−0.0107 (17)
C7	0.0657 (19)	0.125 (3)	0.0575 (18)	0.0086 (19)	0.0119 (16)	−0.0106 (18)
C8	0.0403 (12)	0.0481 (13)	0.0452 (13)	−0.0006 (10)	0.0047 (10)	0.0019 (10)
C9	0.0468 (14)	0.0541 (14)	0.083 (2)	−0.0065 (11)	0.0022 (14)	0.0118 (14)
C10	0.092 (2)	0.082 (2)	0.091 (2)	−0.0002 (18)	0.042 (2)	0.0094 (19)
C11	0.0457 (13)	0.0448 (12)	0.0417 (12)	0.0060 (10)	0.0058 (10)	0.0057 (10)
C12	0.0460 (13)	0.0597 (15)	0.0631 (16)	0.0036 (12)	0.0022 (12)	−0.0096 (12)
C13	0.0440 (14)	0.0769 (18)	0.0763 (19)	0.0044 (13)	0.0054 (14)	0.0058 (16)
C14	0.0548 (16)	0.0751 (18)	0.085 (2)	0.0171 (14)	0.0291 (16)	0.0245 (17)
C15	0.087 (2)	0.0697 (19)	0.079 (2)	0.0193 (17)	0.0405 (19)	0.0017 (16)
C16	0.0689 (17)	0.0584 (15)	0.0520 (15)	−0.0008 (13)	0.0132 (13)	−0.0049 (12)

### Geometric parameters (Å, °)

**Table:**

S1—O2	1.428 (2)	C6—H6	0.9300
S1—O1	1.4371 (18)	C7—H7	0.9300
S1—N1	1.633 (2)	C8—H8	0.9300
S1—C5	1.755 (3)	C9—C10	1.510 (5)
N1—C8	1.301 (3)	C9—H9A	0.9700
N2—C8	1.326 (3)	C9—H9B	0.9700
N2—C11	1.441 (3)	C10—H10A	0.9600
N2—C9	1.467 (3)	C10—H10B	0.9600
C1—C2	1.517 (4)	C10—H10C	0.9600
C1—H1A	0.9600	C11—C12	1.369 (3)
C1—H1B	0.9600	C11—C16	1.379 (4)
C1—H1C	0.9600	C12—C13	1.377 (4)
C2—C7	1.363 (4)	C12—H12	0.9300
C2—C3	1.373 (4)	C13—C14	1.361 (4)
C3—C4	1.376 (4)	C13—H13	0.9300
C3—H3	0.9300	C14—C15	1.353 (4)
C4—C5	1.374 (3)	C14—H14	0.9300
C4—H4	0.9300	C15—C16	1.381 (4)
C5—C6	1.371 (4)	C15—H15	0.9300
C6—C7	1.376 (4)	C16—H16	0.9300
			
O2—S1—O1	117.23 (12)	N1—C8—N2	122.7 (2)
O2—S1—N1	107.27 (12)	N1—C8—H8	118.6
O1—S1—N1	112.35 (10)	N2—C8—H8	118.6
O2—S1—C5	107.76 (11)	N2—C9—C10	111.4 (2)
O1—S1—C5	107.95 (12)	N2—C9—H9A	109.3
N1—S1—C5	103.32 (11)	C10—C9—H9A	109.3
C8—N1—S1	116.09 (17)	N2—C9—H9B	109.3
C8—N2—C11	119.30 (19)	C10—C9—H9B	109.3
C8—N2—C9	121.8 (2)	H9A—C9—H9B	108.0
C11—N2—C9	118.42 (19)	C9—C10—H10A	109.5
C2—C1—H1A	109.5	C9—C10—H10B	109.5
C2—C1—H1B	109.5	H10A—C10—H10B	109.5
H1A—C1—H1B	109.5	C9—C10—H10C	109.5
C2—C1—H1C	109.5	H10A—C10—H10C	109.5
H1A—C1—H1C	109.5	H10B—C10—H10C	109.5
H1B—C1—H1C	109.5	C12—C11—C16	120.1 (2)
C7—C2—C3	118.3 (3)	C12—C11—N2	119.6 (2)
C7—C2—C1	121.3 (3)	C16—C11—N2	120.3 (2)
C3—C2—C1	120.4 (3)	C11—C12—C13	119.2 (3)
C2—C3—C4	121.2 (3)	C11—C12—H12	120.4
C2—C3—H3	119.4	C13—C12—H12	120.4
C4—C3—H3	119.4	C14—C13—C12	120.9 (3)
C5—C4—C3	119.7 (3)	C14—C13—H13	119.5
C5—C4—H4	120.2	C12—C13—H13	119.5
C3—C4—H4	120.2	C15—C14—C13	119.9 (3)
C6—C5—C4	119.6 (3)	C15—C14—H14	120.0
C6—C5—S1	120.3 (2)	C13—C14—H14	120.0
C4—C5—S1	120.1 (2)	C14—C15—C16	120.5 (3)
C5—C6—C7	119.7 (3)	C14—C15—H15	119.8
C5—C6—H6	120.2	C16—C15—H15	119.8
C7—C6—H6	120.2	C11—C16—C15	119.4 (3)
C2—C7—C6	121.6 (3)	C11—C16—H16	120.3
C2—C7—H7	119.2	C15—C16—H16	120.3
C6—C7—H7	119.2		
			
O2—S1—N1—C8	−125.18 (19)	C5—C6—C7—C2	−1.5 (5)
O1—S1—N1—C8	5.1 (2)	S1—N1—C8—N2	−178.33 (18)
C5—S1—N1—C8	121.1 (2)	C11—N2—C8—N1	−173.9 (2)
C7—C2—C3—C4	−0.9 (5)	C9—N2—C8—N1	−1.9 (4)
C1—C2—C3—C4	−179.5 (3)	C8—N2—C9—C10	−96.1 (3)
C2—C3—C4—C5	0.6 (5)	C11—N2—C9—C10	76.0 (3)
C3—C4—C5—C6	−0.7 (4)	C8—N2—C11—C12	55.5 (3)
C3—C4—C5—S1	177.3 (2)	C9—N2—C11—C12	−116.7 (3)
O2—S1—C5—C6	154.6 (2)	C8—N2—C11—C16	−124.7 (3)
O1—S1—C5—C6	27.1 (3)	C9—N2—C11—C16	63.1 (3)
N1—S1—C5—C6	−92.1 (2)	C16—C11—C12—C13	0.3 (4)
O2—S1—C5—C4	−23.4 (2)	N2—C11—C12—C13	−179.9 (2)
O1—S1—C5—C4	−150.9 (2)	C11—C12—C13—C14	0.2 (4)
N1—S1—C5—C4	89.9 (2)	C12—C13—C14—C15	0.2 (5)
C4—C5—C6—C7	1.1 (5)	C13—C14—C15—C16	−1.0 (5)
S1—C5—C6—C7	−176.9 (3)	C12—C11—C16—C15	−1.2 (4)
C3—C2—C7—C6	1.3 (5)	N2—C11—C16—C15	179.1 (2)
C1—C2—C7—C6	179.9 (3)	C14—C15—C16—C11	1.5 (4)
